# Posterior cingulate cortex hyperactivity in conversion disorder: a PET/MRI study

**DOI:** 10.3389/fpsyt.2024.1336881

**Published:** 2024-03-07

**Authors:** Safiye Zeynep Tatlı, Mine Araz, Elgin Özkan, Elif Peker, Mehmetİlhan Erden, VesileŞentürk Cankorur

**Affiliations:** Faculty of Medicine, Ankara University, Ankara, Türkiye

**Keywords:** posterior cingulate cortex, conversion disorder, glucose metabolism, neuroimaging, PET/MRI

## Abstract

**Introduction:**

Several neuroimaging studies have been conducted to demonstrate the specific structural and functional brain correlations of conversion disorder. Although the findings of neuroimaging studies are not consistent, when evaluated as a whole, they suggest the presence of significant brain abnormalities. The aim of this study is to investigate brain metabolic activity through F-18 fluorodeoxyglucose PET/MRI in order to shed light on the neural correlates of conversion disorder.

**Methods:**

20 patients diagnosed with conversion disorder were included in the study. Hamilton Depression and Anxiety Rating Scales, Somatosensory Amplification Scale and Somatoform Dissociation Scale were administered. Then, brain F-18 FDG-PET/MRI was performed..

**Results:**

Hypermetabolism was found in posterior cingulate R, while glucose metabolisms of other brain regions were observed to be within the normal limits. When compared with the control group, statistically significant differences in z-scores were observed among all brain regions except for parietal superior R and cerebellum. No correlation was observed between the metabolisms of the left ACC and left medial PFC; left ACC and left temporal lateral cortex; cerebellum and left parietal inferior cortex despite the presence of positive correlations between these regions in the opposite hemisphere.

**Discussion:**

Results of the study suggest a potential involvement of the DMN which is associated with arousal and self-referential processing as well as regions associated with motor intention and self-agency.

## 1 Introduction

Psychosocial factors have been emphasized in the etiology of conversion disorder historically. Higher frequency of conversion symptoms in individuals with neurological diseases, females, and on the left side of the body suggests the involvement of biological factors (1, 2). Several neuroimaging studies have demonstrated specific structural and functional brain correlations however the findings are not consistent (2–5). No study with PET/MRI on conversion disorder has been performed to the best of our knowledge to date. The aim of this study is to investigate brain metabolic activity through F-18 FDG PET/MRI in order to shed light on the neural correlates of conversion disorder.

## 2 Materials and methods

A total of 20 patients who presented to the outpatient clinic, aged between 18-65 years, who were literate, examined by at least one neurologist and psychiatrist, evaluated for neurological and other medical conditions and diagnosed with conversion disorder according to DSM-5 diagnostic criteria, were voluntarily included in the study. Neurological examinations of all patients were normal and their symptoms of conversion disorder were active. All patients were found to be right-hand dominant.

Exclusion criteria included intellectual disability, a history of neurological disease or traumatic brain injury, presence of movement symptoms at rest and presence of a current or previous DSM-5 diagnosis of any major mental disorder other than anxiety disorders or depression. The study was conducted after obtaining approval from the Human Research Ethics Committee (I2-108-20). Informed Consent Forms were signed by the participants prior to the study.

Patients underwent neurological and psychiatric evaluations. After the Structured Clinical Interview for DSM-5 (SCID-5-CV), Hamilton Depression Rating Scale (HDRS), and Hamilton Anxiety Rating Scale (HARS) were administered by the first co-author, the patients were asked to complete the Sociodemographic and Clinical Information Form, Somatosensory Amplification Scale (SSAS), Somatoform Dissociation Scale (SDQ-20) and Edinburgh Handedness Inventory (EHI).

### 2.1 PET/MRI imaging protocol

Brain PET/MR imaging was performed with a simultaneous PET/MRI system (3 Tesla, SIGNA PET/MR, GE Healthcare) at the Department of Nuclear Medicine. Patients who were taking psychotropic medication were asked not to take medication for at least 24 hours prior to the imaging. Patients continued their regular treatment after the imaging. Furthermore, they were instructed to refrain from strenuous activities and consumption of caffeine-containing products for 24 hours. Patients fasted at least 6 h before imaging and blood glucose levels were checked. Those with a blood glucose level above 150 mg/dl did not undergo scanning. The patient was equipped with earplugs and had eyes covered, after which approximately 210 MBq F-18 FDG was injected intravenously. Sixty minutes after tracer injection a single-bed 20 min MRI acquisition and PET emission started at the same time and all MRI sequences were completed within the duration of the PET scan.

The MRI protocol included sagital 3D T2 FLAIR images (matrix:512x512, slice thickness:1.2 mm, slice gap:0.6 mm, TR:6500 msec, TE:112 msec, ETL: 200, flip angle:90°, slice number:312, inversion time:1700), axial diffusion weighted images (matrix:256x256, slice thickness:4 mm, slice gap:5 mm, TR:4400 msec, TE:112 msec, b-value:1000, slice number:32), axial 3D T2* SWAN (matrix:512x512, slice thickness:2,2 mm, slice gap:1,1 mm, TR:38 msec, slice number:148), 3D T1 image (matrix:512x512, slice thickness:1.2 mm, slice gap:0.6 mm, TR:8 msec, TE:2,9 msec, flip angle:12°, slice number:268, inversion time:550), axial T2 FLAIR (matrix:512x512, slice thickness:4 mm, slice gap:5 mm, TR:8800 msec, TE:137 msec, ETL:27, flip angle:160°, slice number:32, inversion time:2400), axial T1 weighted images (matrix:512x512, slice thickness:4 mm, slice gap:5 mm, TR:400 msec, TE:10 msec, ETL:4, flip angle:110°, slice number:32), axial T2 weighted images (matrix:512x512, slice thickness:4 mm, slice gap:5 mm, TR:4800 msec, TE:106 msec, ETL:32, flip angle:142°, slice number:32), coronal T2 weighted images (matrix:512x512, slice thickness:4 mm, slice gap:5 mm, TR:7200 msec, TE:113 msec, ETL:28, flip angle:111°, slice number:32).

### 2.2 Data processing

**Figure 1:**
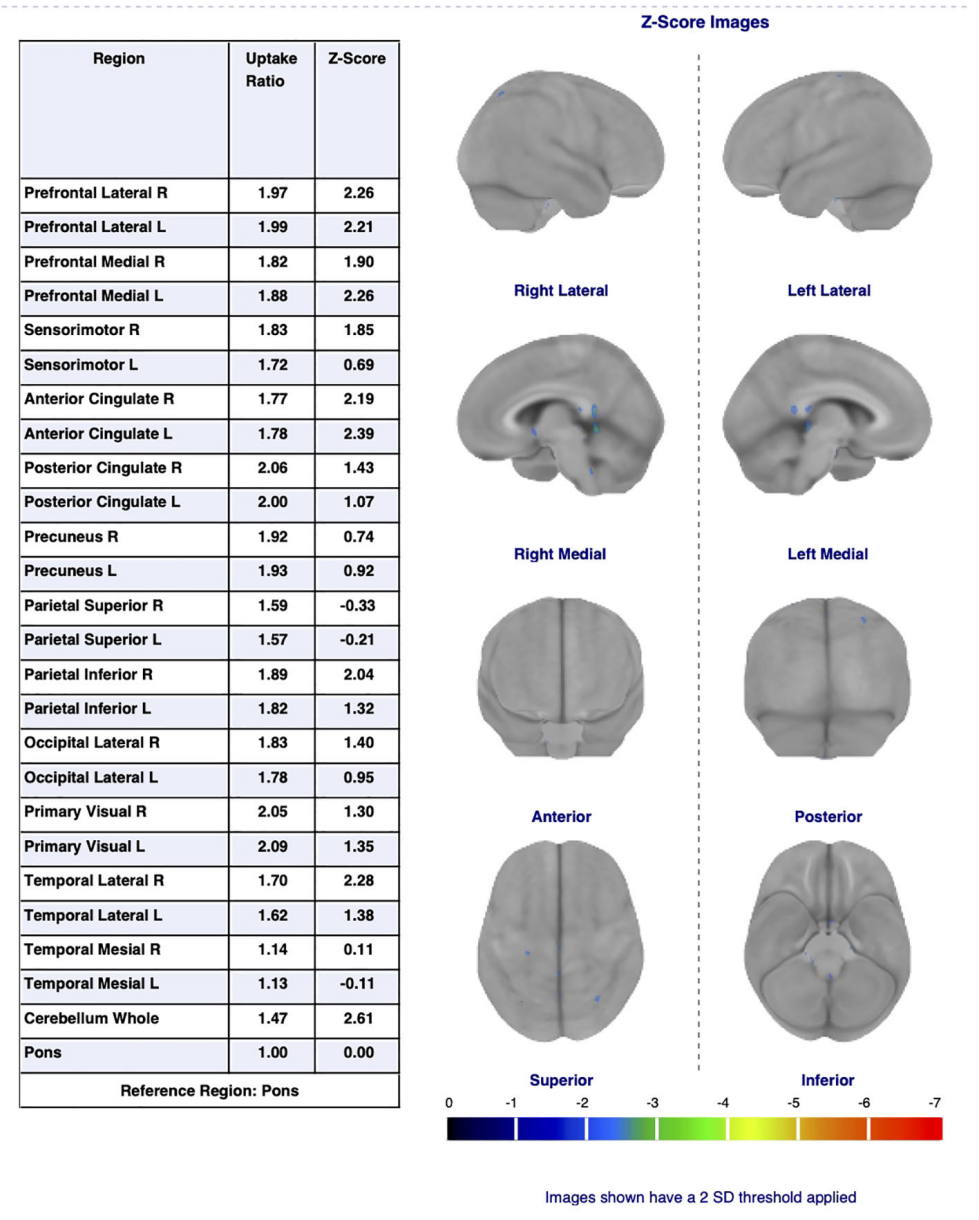
F-18 FDG uptake rates, z-scores, and color-coded images of a patient.

**Figure 2:**
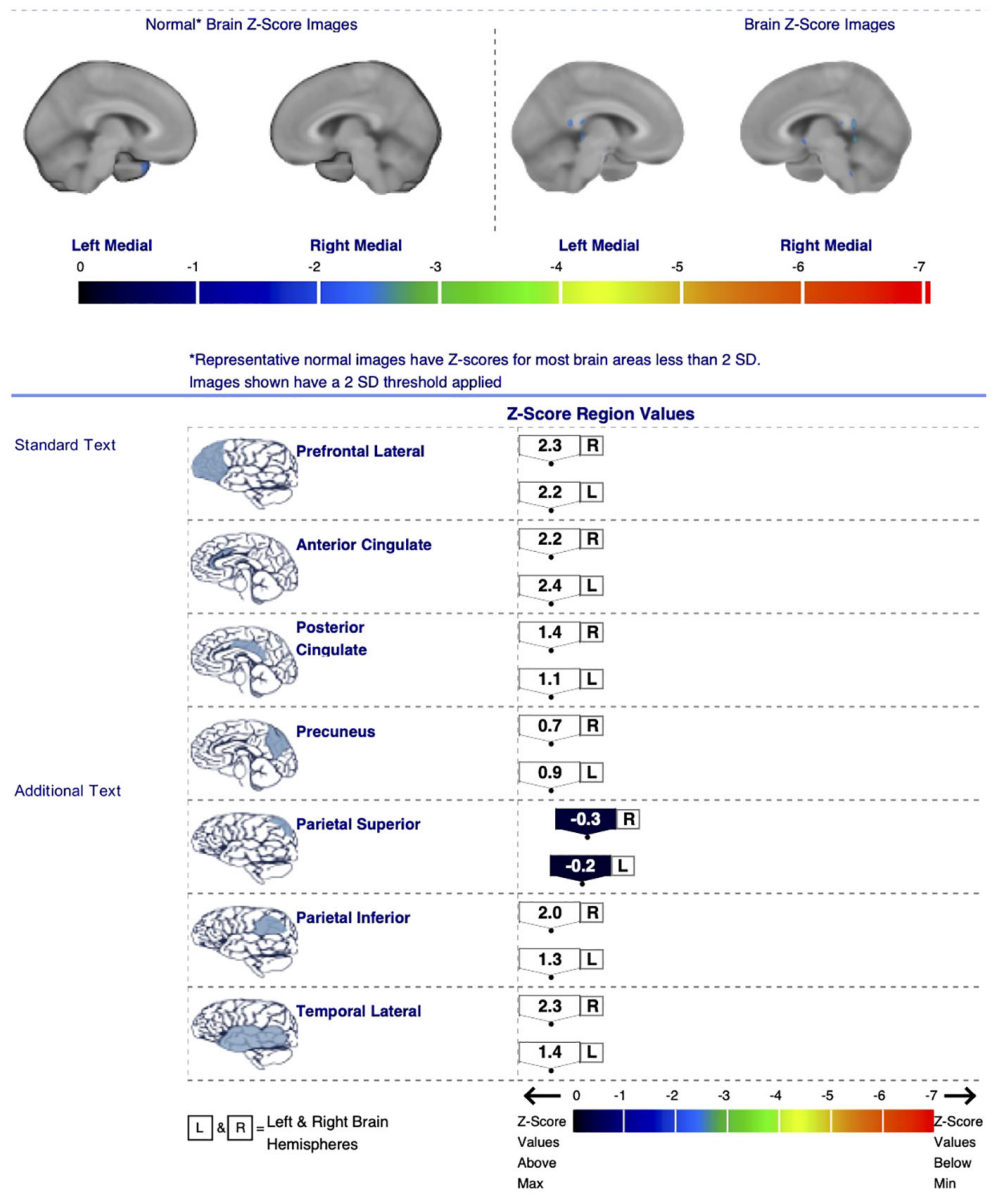
Color-coded images of a patient (right), an age-matched healthy control (left) and a visual summary of z-scores of the patient’s brain regions (bottom).

A total of 21 different regions were evaluated for each patient, including the bilateral prefrontal lateral, prefrontal medial, sensorimotor, anterior cingulate (ACC), posterior cingulate (PCC), precuneus, superior parietal, inferior parietal, lateral temporal, mesial temporal cortices, and the entire cerebellum. Age-matched healthy control data for each region were obtained from the device’s database, and z-scores representing the number of standard deviations were determined. The results were interpreted with visual inspection, regional glucose metabolic rates and z-scores. A z-score of +2 or above indicated hypermetabolism, while a z-score of -2 or below indicated hypometabolism in a particular region (6–8). **Figure 1** shows the F-18 FDG uptake rates, z-scores, and color-coded images of a patient participating in the study, as an example. **Figure 2** shows color-coded images of the same patient, an age-matched healthy control and a visual summary of z-scores of the patient’s brain regions.

### 2.3 Statistical analysis

SPSS 11.5 package program (IBM Corp, Armonk, NY) was used for data analysis. Descriptive statistics such as mean ± standard deviation and median (minimum-maximum) were used for quantitative variables, while the number of patients (percentage) was used for qualitative variables. The impact of clinical risk factors on z-scores was assessed using multivariate linear regression analysis. To determine the direction and strength of the correlation between continuous variables, Spearman’s correlation analysis was used. The statistical significance level was set as 0.05.

## 3 Results

The mean age of the patients (n=20) was 30.3 ± 7.7, and 75.0% of them were female, 45.0% of the patients were married and had children. The mean duration of education was 10.9 ± 3.7 years and 30.0% of the patients were employed. 50.0% of the patients were smokers. None of the patients had alcohol or substance use disorder. 35.0% of the patients had an additional physical illness (diabetes mellitus, n=3; hypertension, n=4).

The mean age of onset of the disorder was 25.7 ± 7.3 years, and the mean total duration of the illness was 4.7 ± 6.1 years. 40.0% of them had a history of childhood trauma, and all reported at least one current stressful life event. Most of the patients had complaints related to multiple symptom areas of conversion disorder; 75.0% experienced fainting, 45.0% had motor symptoms, and 25.0% had sensory symptoms. 35.0% of the patients had suicidal thoughts and 25.0% had a history of at least one suicide attempt during their lifetime. 30.0% of the patients had a family history of a similar illness, and 50.0% had a family history of mental illness. 70.0% had la belle indifference. 40.0% of the patients were not receiving any psychopharmacological treatment at the time of inclusion, while the others were using an antidepressant, a mood stabilizer, or a combination of these medications.

Upon examining the data from the clinical assessment scales, the mean scores were 21.5 ± 10.0 for HDRS, 22.6 ± 8.8 for HARS, 33 ± 6.9 for SSAS and 37.4 ± 11.5 for SDQ-20 (**Table 1**).

**Table 1:** Descriptive statistics for demographic and clinical variables.

Variables		
**Age**	Mean ± SD	30.3 ± 7.7
	Median (Min-Max)	28.0 (19.0-41.00)
**Gender, n(%)**	Female	15 (75.0)
	Male	5 (25.0)
**Marital Status, n(%)**	Single	11 (55.0)
	Married	9 (45.0)
**Children, n(%)**	No	11 (55.0)
	Yes	9 (45.0)
**Education, years**	Mean ± SD	10.9 ± 3.7
	Median (Min-Max)	12 (5.0-16.0)
**Employment status, n(%)**	Unemployed	14 (70.0)
	Employed	6 (30.0)
**Chronic illness, n(%)**	No	13 (65.0)
	Yes	7 (35.0)
**Tobacco use, n(%)**	No	10 (50.0)
	Yes	10 (50.0)
**Illness onset, age in years**	Mean ± SD	25.7 ± 7.3
	Median (Min-Max)	24.5 (15.0-37.0)
**Illness duration, years**	Mean ± SD	4.7 ± 6.1
	Median (Min-Max)	2.5 (1.0-23.0)
**Childhood trauma, n(%)**	No	12 (60.0)
	Yes	8 (40.0)
**Fainting, n(%)**	No	5 (25.0)
	Yes	15 (75.0)
**Motor symptoms, n(%)**	No	11 (55.0)
	Yes	9 (45.0)
**Sensory symptoms, n(%)**	No	15 (75.0)
	Yes	5 (25.0)
**Suicidal thoughts, n(%)**	No	13 (65.0)
	Yes	7 (35.0)
**Suicide attempt, n(%)**	No	15 (75.0)
	Yes	5 (25.0)
**Family history of similar illness, n(%)**	No	14 (70.0)
	Yes	6 (30.0)
**Family history of mental illness, n(%)**	No	10 (50.0)
	Yes	10 (50.0)
**La belle indifference, n(%)**	No	6 (30.0)
	Yes	14 (70.0)
**HDRS score**	Mean ± SD	21.5 ± 10.0
	Median (Min-Max)	21.0 (1.0-39.0)
**HARS score**	Mean ± SD	22.6 ± 8.8
	Median (Min-Max)	24.5 (0.0-36.0)
**SSAS score**	Mean ± SD	33.0 ± 6.9
	Median (Min-Max)	33.5 (13.0-44.0)
**SDQ-20 score**	Mean ± SD	37.4 ± 11.5
	Median (Min-Max)	35.5 (20.0-69.0)

HDRS, Hamilton Depression Rating Scale, HARS, Hamilton Anxiety Rating Scale, SSAS, Somatosensory Amplification Scale, SDQ, Somatoform Dissociation Questionnaire.

For all brain regions examined, the mean z-score values of the patients were found to be within the normal range, except for posterior cingulate R, where mild hypermetabolism was observed. Neither hypometabolism nor hypermetabolism was detected in the other examined brain regions. When compared with the control group, statistically significant differences in z-scores were observed among all brain regions except for parietal superior R and cerebellum (**Table 2**).

**Table 2:** Comparative analysis of the Z scores between control and patient groups.

Regions	Group				*P* value
	Healthy Control		Patient		
	Mean ± SD	Median (Min-Max)	Mean ± SD	Median (Min-Max)	
**Prefrontal Lateral R**	1.71 ± 0.03	1.72 (1.58-1.72)	1.89 ± 0.16	1.90 (1.51-2.24)	**<0.001^a^**
**Prefrontal Lateral L**	1.72 ± 0.04	1.73 (1.57-1.73)	1.86 ± 0.18	1.85 (1.39-2.17)	**<0.001^a^**
**Prefrontal Medial R**	1.60 ± 0.04	1.61 (1.45-1.61)	1.71 ± 0.15	1.68 (1.42-2.02)	**<0.001^a^**
**Prefrontal Medial L**	1.61 ± 0.04	1.62 (1.46-1.62)	1.73 ± 0.17	1.71 (1.35-2.06)	**<0.001^a^**
**Sensorimotor R**	1.63 ± 0.03	1.64 (1.51-1.64)	1.77 ± 0.17	1.74 (1.54-2.18)	**0.004^a^**
**Sensorimotor L**	1.64 ± 0.03	1.65 (1.51-1.65)	1.73 ± 0.16	1.72 (1.43-2.11)	**0.003^a^**
**Anterior Cingulate R**	1.47 ± 0.04	1.49 (1.32-1.49)	1.60 ± 0.18	1.55 (1.26-1.99)	**0.017^a^**
**Anterior Cingulate L**	1.46 ± 0.04	1.48 (1.30-1.48)	1.57 ± 0.25	1.55 (0.82-2.02)	**<0.001^a^**
**Posterior Cingulate R**	1.85 ± 0.04	1.86 (1.68-1.86)	2.03 ± 0.17	2.03 (1.68-2.40)	**<0.001^a^**
**Posterior Cingulate L**	1.84 ± 0.04	1.85 (1.67-1.85)	1.92 ± 0.38	2.00 (0.45-2.32)	**0.001^a^**
**Precuneus R**	1.81 ± 0.03	1.82 (1.68-1.82)	1.91 ± 0.16	1.92 (1.53-2.33)	**0.001^a^**
**Precuneus L**	1.80 ± 0.03	1.81 (1.66-1.81)	1.93 ± 0.16	1.92 (1.60-2.33)	**<0.001^a^**
**Parietal Superior R**	1.62 ± 0.04	1.63 (1.48-1.63)	1.66 ± 0.32	1.68 (0.62-2.03)	0.284^a^
**Parietal Superior L**	1.59 ± 0.03	1.60 (1.45-1.60)	1.71 ± 0.19	1.71 (1.37-2.11)	**0.008^a^**
**Parietal Inferior R**	1.66 ± 0.03	1.67 (1.54-1.67)	1.81 ± 0.16	1.76 (1.48-2.13)	**<0.001^a^**
**Parietal Inferior L**	1.66 ± 0.03	1.67 (1.53-1.67)	1.75 ± 0.17	1.75 (1.37-2.10)	**0.003^a^**
**Temporal Lateral R**	1.48 ± 0.02	1.49 (1.39-1.49)	1.59 ± 0.12	1.60 (1.40-1.82)	**0.001^a^**
**Temporal Lateral L**	1.48 ± 0.02	1.49 (1.39-1.49)	1.56 ± 0.13	1.55 (1.30-1.83)	**0.008^a^**
**Temporal Mesial R**	1.12 ± 0.02	1.13 (1.06-1.13)	1.18 ± 0.06	1.16 (1.09-1.33)	**0.001^a^**
**Temporal Mesial L**	1.13 ± 0.02	1.14 (1.06-1.14)	1.19 ± 0.08	1.17 (1.11-1.44)	**0.017^a^**
**Cerebellum**	1.29 ± 0.01	1.29 (1.25-1.29)	1.34 ± 0.11	1.31 (1.20-1.52)	0.624^a^
**Pons**	1.00 ± 0.00	1.00 (1.00-1.00)	1.00 ± 0.00	1.00 (1.00-1.00)	1.000^a^

R, Right; L, Left; SD, Standard deviation; Min, Minimum; Max, Maximum; ^a^: Mann Whitney U test.

Bold p-values indicate statistically significant findings (p < 0.05).

Correlation analyses were conducted among brain regions’ z-scores. The results of the analysis are presented in **Table 3**. Repetitive data are indicated once.

**Table 3:** Correlation of z-scores among brain regions.

	PFL-R	PFL-L	PFM-R	PFM-L	SM-R	SM-L	ACC-R	ACC-L	PCC-R	PCC-L	PreC-R	PreC-L	PS-R	PS-L	PI-R	PI-L	TL-R	TL-L	TM-R	TM-L	Cer
**PFL-R**	1																				
**PFL-L**	.72†	1																			
**PFM-R**	.86†	.70†	1																		
**PFM-L**	.73†	.76†	.82†	1																	
**SM-R**	.80†	.59†	.73†	.71†	1																
**SM-L**	.77†	.81†	.76†	.83†	.79†	1															
**ACC-R**	.54*	.51*	.59†	.41	.36	.33	1														
**ACC-L**	.62†	.34	.68†	.42	.33	.34	.67†	1													
**PCC-R**	.39†	.41	.50*	.60†	.51*	.64†	-.00	.28	1												
**PCC-L**	.61†	.66†	.73†	.82†	.60†	.79†	.19	.38	.79†	1											
**PreC-R**	.57†	.63†	.45*	.51*	.50*	.68†	.17	.04	.43	.52*	1										
**PreC-L**	.74†	.59†	.74†	.77†	.74†	.81†	.25	.32	.61†	.73†	.80†	1									
**PS-R**	.65†	.59†	.45*	.43	.67†	.54*	.37	.17	.11	.22	.64†	.62†	1								
**PS-L**	.54*	.60†	.50*	.51*	.60†	.63†	.35	.25	.35	.47*	.55*	.58†	.75†	1							
**PI-R**	.76†	.46*	.70†	.66†	.79†	.63†	.25	.27	.24	.53*	.50*	.76†	.59†	.35	1						
**PI-L**	.51*	.73†	.58†	.74†	.63†	.88†	.21	.15	.69†	.78†	.67†	.77†	.49*	.69†	.45*	1					
**TL-R**	.72†	.48*	.73†	.63†	.64†	.59†	.48*	.57†	.36	.61†	.41	.72†	.38	.29	.81†	.46*	1				
**TL-L**	.54*	.86†	.57†	.65†	.53*	.79†	.31	.23	.53*	.66†	.67†	.61†	.43	.51*	.31	.81†	.37	1			
**TM-R**	.70†	.51*	.81†	.66†	.61†	.54*	.62†	.61†	.29	.54*	.36	.61†	.51*	.66†	.60†	.38	.64†	.25	1		
**TM-L**	.77†	.62†	.81†	.71†	.59†	.53*	.68†	.71†	.31	.56*	.34	.56*	.43	.36	.66†	.31	.80†	.36	.83†	1	
**Cer**	.55*	.40	.62†	.66†	.33	.46*	.39	.45*	.19	.48*	.18	.43	.08	-.00	.55*	.25	.62†	.28	.44	.64†	1

PFL-R, Prefrontal lateral right; PFL-L, Prefrontal lateral left; PFM-R, Prefrontal medial right; PFM-L, Prefrontal medial left; SM-R, Sensorimotor right; SM-L, Sensorimotor left; ACC-R, Anterior cingulate cortex right; ACC-L, Anterior cingulate cortex; PCC-R, Posterior cingulate cortex right; PCC-L, Posterior cingulate cortex left; PreC-R, Precuneus right; PreC-L, Precuneus left; PS-R, Parietal superior right; PS-L, Parietal superior left; PI-R, Parietal inferior right; PI-L, Parietal inferior left; TL-R, Temporal lateral right; TL-L, Temporal lateral left; TM-R, Temporal mesial right; TM-L, Temporal mesial left; Cer, Cerebellum.

*Correlation is significant at the 0.05 level, †Correlation is significant at the 0.01 level.

The different color shading represents statistically significant findings.

Multivariate linear regression analyzes were performed to examine the relationship between the z-scores of the brain regions and the clinical features of the patients (la belle indifference, childhood trauma and duration of illness). As a result of the analysis for the Prefrontal Lateral L Z score, duration of illness and la belle indifference was found to be significant factors. In the presence of other variables, an increase of one year in the duration of illness led to a decrease of 0.082 units, and the presence of la belle indifference resulted in an increase of 1.011 units in the Prefrontal Lateral L Z score. Furthermore, the two variables together accounted for 34.6% of the variation in the Prefrontal Lateral L Z score. As a result of the analysis for the Anterior Cingulate R, Anterior Cingulate L, Temporal Mesial R and Temporal Mesial L Z scores, duration of illness was found to be a significant factor. An increase of one year in the duration of illness resulted in a decrease of 0.089, 0.096, 0.059 and 0.052 units in the Anterior Cingulate R, Anterior Cingulate L, Temporal Mesial R and Temporal Mesial L Z scores, respectively. Illness duration alone explained 27.5%, 33.4%, 22.8% and 19.9% of the variation in the Anterior Cingulate R, Anterior Cingulate L, Temporal Mesial R and Temporal Mesial L Z scores, respectively. Z scores of Prefrontal Lateral R, bilateral Prefrontal medial, Sensorimotor, Parietal Superior, Parietal Inferior, Temporal Lateral cortices and cerebellum yielded non-significant results for all clinical variables when analyzed together (p>0.05) (**Table 4**).

**Table 4:** Results of multivariate linear regression analysis.

Regions	Variables	β	SE	*P* value	R^2^	%95 CI for B (Lower Bound–Upper Bound)
**Prefrontal Lateral L Z score**	**Constant**	-0.854	0.852	0.331	0.346	-2.652-0.945
	**Duration of illness**	-0.082	0.037	0.041		-0.160-(-0.004)
	**La belle indifference**	1.011	0.478	0.050		0.002-2.019
**Anterior Cingulate R Z score**	**Constant**	0.992	0.258	0.001	0.275	0.450-1.535
	**Duration of illness**	-0.089	0.034	0.018		-0.161-(-0.017)
**Anterior Cingulate L Z score**	**Constant**	1.237	0.241	<0.001	0.334	0.731-1.742
	**Duration of illness**	-0.096	0.032	0.008		-0.163-(-0.029)
**Temporal Mesial R Z score**	**Constant**	0.875	0.193	<0.001	0.228	0.470-1.280
	**Duration of illness**	-0.059	0.026	0.033		-0.113-(-0.005)
**Temporal Mesial L Z score**	**Constant**	0.894	0.185	<0.001	0.199	0.506-1.282
	**Duration of illness**	-0.052	0.024	0.049		-0.103-(-0.001)

SE, Standard error; CI, Confidence interval.

## 4 Discussion

The results of this study have indicated hypermetabolism in posterior cingulate R, while glucose metabolisms of other brain regions were observed to be within the normal limits. In the literature, only one study utilizing FDG-PET in patients with conversion disorder has been identified. According to the findings of this study, hypometabolism in the right inferior parietal region and bilateral ACC was reported (9). It is considered that the observed differences in the results may be attributed to three main reasons: first, aforementioned study included only patients with seizure-like symptoms; second, these were severe patients who had intractable seizures and required inpatient assessment in a specialized epilepsy unit; third, the presence of psychiatric comorbidity, a potential confounding factor, was not assessed. Studies conducted using fMRI have demonstrated increased activity in the PCC and deep limbic system structures. This phenomenon has been attributed to preferential activation of regions associated with emotional processing, rather than sensory-motor functions (10–12). In our study, hypermetabolism was also detected in PCC. PCC is known as a central node in the DMN which is active when attention is directed internally (i.e., toward body movement). There is mounting evidence of PCC playing a crucial role in controlling the state of arousal and the internal or external focus of attention (13). In addition to DMN, PCC is also a part of in frontoparietal control network and involved in executive motor control. There are two interpretations of this result; firstly, the hypermetabolism in PCC may be reflecting the patients’ increased efforts in arousal and self-referential processing, secondly it may represent the inability to deactivate PCC which leads to problems with cognitive flexibility, focus of attention, noticing internal and external changes, facilitating novel behavior in response and motor control (14). When compared with the control group, statistically significant differences in Z scores were observed among all brain regions except parietal superior R and the cerebellum. Considering all these results together, it can be stated that there are widespread metabolic changes in many brain regions compared to healthy controls, in addition to hypermetabolism in PCC. Therefore, instead of looking for a specific region associated with conversion disorder, conducting correlational or connectivity studies to determine related pathways can be more useful.

### 4.1 Correlations

It has been observed that the identified 9 regions (PFK-L, PFK-M, SM, ACC, PCC, PreC, PS, PI and TM) have moderate-high positive correlations between homologous regions in the two hemispheres. TL regions did not have significant homologous associativity, suggesting more specific lateralized function. Consistent findings have been reported in studies investigating metabolic correlations between homologous brain regions in healthy adults (15).

It is noteworthy that no correlation was observed between the metabolisms of the left ACC and left medial PFC; left ACC and left temporal lateral cortex; cerebellum and left parietal inferior cortex despite the presence of positive correlations between these regions in the opposite hemisphere. There are studies that associate conversion disorder with structural and functional changes in medial PFC (16, 17), ACC (4, 18), temporal cortex (19), inferior parietal cortex (9, 20) and cerebellum (9, 21). Recent meta-analyses conducted in this field have also associated changes in the ACC and temporal cortex metabolisms (3), and alterations in both the structure and functions of the ventromedial PFC and ACC with conversion disorder (5). Medial PFC, lateral temporal cortex, ACC as well as inferior parietal areas are considered core regions associated with the Default Mode Network (DMN), which is a functional network in the brain that is involved in self-reflection, autobiographical memory and social cognition (22–24). Several studies have suggested that disruptions or altered connectivity within the DMN may play a role in the development and expression of conversion disorder symptoms (25–28). In our study, the lack of correlation between the left ACC and left medial PFC; left ACC and left temporal lateral cortex; left parietal inferior cortex and cerebellum contrary to the homologous hemisphere may suggest a potential association of the DMN and conversion disorder. Disruption within the DMN can affect the coordination of cognitive, emotional, and sensory processes, potentially leading to the conversion symptoms.

Left inferior parietal cortex is a convergence zone of various brain networks, which plays important roles in attention, language, social cognition and suggested as a critical area involved in the integration of the self-consciousness of the body (29). And for the cerebellum; in addition to its well-known role in motor coordination and motor learning, emerging evidence indicates its involvement in cognitive processes, emotion regulation, sensory integration and those associated with the DMN such as self-referential processing, introspection, and mind-wandering (30). These functions could be relevant to the understanding of conversion disorder as one of the hypothesis underlying conversion disorder is dysfunctional motor intention and loss of self agency. In a PET study conducted with conversion disorder patients exhibiting seizure-like symptoms, increased metabolic correlation between the right inferior parietal cortex and cerebellum was reported (9). Moreover there are several studies that show temporoparietal hypoactivation using fMRI in patients with motor symptoms (12, 31). The connections between these regions seem to facilitate the exchange of information related to motor control, spatial processing, and sensory integration and contribute to sensorimotor processing, spatial awareness, and other cognitive functions. Changes in the correlation between the cerebellum and parietal inferior cortex (such as the absence of left-sided correlation or the presence of right-sided correlation) may be associated with the disruption of sensorimotor processing and sensory integration, potentially leading to the manifestation of symptoms in conversion disorder.

### 4.2 Association of neuroimaging findings with clinical features

#### 4.2.1 Left PFC lateral – La belle indifference

The metabolism of the left lateral PFC has been found to be positively associated with the presence of la belle indifference. Patients with la belle indifference, despite experiencing functional impairments, may appear significantly less distressed or indifferent towards conversion symptoms and their consequences. This phenomenon may be associated with the cognitive-emotional interactions or impairment of emotion regulation mechanisms. The lateral PFC contributes to the cognitive-emotional interactions by suppressing or enhancing emotional responses controlled by the amygdala (32). Emotion regulation refers to the process by which individuals modify the attention to emotional information and intensity of emotional experience. One of the most important strategies of emotion regulation is cognitive reappraisal, which involves reinterpreting the meaning of a situation to adjust its emotional impact (33, 34). Numerous studies have associated cognitive reappraisal with the lateral prefrontal cortex (32, 35) and the left lateral prefrontal cortex specifically (35, 36), supporting the proposal that cognitive-control related functions of the lateral PFC down-regulate the negative emotional information (37). Taken together, the left lateral PFC metabolism may contribute to impaired emotional processing resulting with the lack of concern or indifference about their own condition.

#### 4.2.2 Left lateral PFC, bilateral ACC and temporal mesial cortices – duration of illness

The metabolisms of left lateral PFC, bilateral ACC and bilateral mesial temporal cortices has been found to be inversely associated with the duration of illness. PFC, as mentioned above, may contribute to the pathophysiology of conversion disorder through abnormal emotional processing or disruptions in top-down regulation. Alterations in PFC and lateral PFC metabolisms have been associated with conversion disorder in numerous studies (3, 5). ACC is a part of the limbic system which is involved in emotion regulation, memory processing, and the integration of emotional and cognitive processes (38). Structural and functional alterations in the ACC have been associated with conversion disorder in various studies (4, 18). The mesial temporal cortex, particularly the hippocampus, is primarily associated with memory functions, such as encoding and retrieval of emotionally salient experiences. In several neuroimaging studies, structural and functional changes in the mesial temporal cortex have been associated with conversion disorder (19, 28). The association of the metabolic changes in lateral PFC, ACC and temporal mesial cortex with the duration of illness may indicate a potential relationship between these regions and the pathophysiology of conversion disorder, and possibly the course of illness. However, further detailed studies are needed in this regard.

## 5 Conclusion

The relationship between the metabolisms of specific brain regions and conversion disorder is likely to be complex and multifactorial, involving interactions with other brain regions and networks. Taken all together, our results suggest a potential involvement of the DMN which is associated with arousal and self-referential processing as well as regions associated with motor intention and self-agency. Factors such as duration of illness, presence of la belle indifference and the individual interplay between cognitive, emotional and motor processes may influence the extent of contribution of specific brain regions to the emergence of conversion disorder.

This study is not only the first to utilize integrated PET/MRI in conversion disorder but also one of the limited number of functional neuroimaging studies in this field. Furthermore, parameters previously unexplored in prior studies, such as clinical features, exaggeration of bodily sensations, somatization and dissociation have been evaluated. The limitations of our study include the inability to assess the activities of deep brain regions such as amygdala and thalamus; the diversity in patients’ symptoms; high scores for anxiety and depression; and the medication usage in some patients, despite being discontinued at least 24 hours before imaging. Further studies are needed to better understand the mechanisms and functional implications of the specific brain regions or, more likely, functional disintegration of brain networks that underlie conversion disorder.

## Data availability statement

The raw data supporting the conclusions of this article will be made available by the authors, without undue reservation.

## Ethics statement

The studies involving humans were approved by Ethics Committee of Ankara University, School of Medicine (I2-108-20). The studies were conducted in accordance with the local legislation and institutional requirements. The participants provided their written informed consent to participate in this study.

## Author contributions

ST: Conceptualization, Data curation, Formal Analysis, Funding acquisition, Investigation, Methodology, Project administration, Resources, Software, Validation, Visualization, Writing – original draft, Writing – review & editing. MA: Conceptualization, Data curation, Formal Analysis, Methodology, Supervision, Writing – review & editing. EO: Conceptualization, Data curation, Methodology, Supervision, Writing – review & editing. EP: Data curation, Methodology, Supervision, Writing – review & editing. ME: Conceptualization, Methodology, Supervision, Writing – review & editing. VC: Conceptualization, Data curation, Funding acquisition, Investigation, Methodology, Project administration, Supervision, Writing – review & editing.
